# Outcomes of Percutaneous Image-Guided and Laparoscopic Cholecystostomies in High-Risk Patients With Acute Calculus Cholecystitis: A Five-Year District General Hospital Experience

**DOI:** 10.7759/cureus.54313

**Published:** 2024-02-16

**Authors:** Mahmoud S Aly, Zohaib Jamal, Zeeshan Khawaja, Phuong L Kieu, Nowera Zafar, Divya Kanakalingam, Ahmed Khalil

**Affiliations:** 1 Department of Surgery, Wrightington, Wigan and Leigh NHS Foundation Trust, Wigan, GBR; 2 Department of Otolaryngology, Wrightington, Wigan and Leigh NHS Foundation Trust, Wigan, GBR; 3 Department of General Surgery, East Lancashire Hospitals NHS Trust, Blackburn, GBR

**Keywords:** comorbidity, highrisk, laparoscopic cholecystectomy, acute cholecysistitis, cholecystostomy

## Abstract

Introduction

Acute cholecystitis (AC), inflammation of the gall bladder, is one of the most common emergency surgical presentations. In the UK, approximately 15% of the population is estimated to have gallstones, and approximately 20% of them can develop AC. Laparoscopic cholecystectomy (LC) is considered the definitive management of AC. However, cholecystectomy carries a very high risk of morbidity and mortality in high-risk frail patients with multiple comorbidities who are deemed unfit for surgery. Percutaneous cholecystostomy (PC), both image-guided and laparoscopic, is generally acknowledged as an interim treatment measure before definitive management, which is the LC.

Materials and methods

This is a retrospective study from the Royal Albert Edward Infirmary, a district general hospital (DGH) based in Wigan, UK. The medical records of all the patients who were admitted to the surgical department and underwent PC between January 2017 and December 2022 were analyzed. Patients with previous hepato-pancreato-biliary (HPB) malignancy, who underwent open cholecystostomy, or those with abdominal ascites were excluded from the study. Information was collected regarding the age, gender, American Society of Anaesthesiologists (ASA) grades, success rates of both procedures as temporary or definitive management, duration of hospital stay, 30-day and 1-year mortality after the procedure, timing of the procedure, and long-term complications after the procedure, particularly those related to cholecystostomy tube dislodgment or blockage.

Results

Twenty-seven patients who underwent PC were divided into two groups: group A, consisting of 10 patients who underwent laparoscopic cholecystostomies, and group B, consisting of 17 patients who had ultrasound (US)-guided cholecystostomies. The mean age of the patients in group A was 66.7 as compared to 75.1 in group B. Most of the patients were in ASA groups III (14) and IV (10). About 74% of patients had procedures done during the day and 26% had PC at night time. The mean hospital stay was 13.5 days. About 55% of patients had planned elective LC as a definitive management. Following the treatment, two patients died within 30 days, and eight patients passed away within a year. About 40% of the patients had complications related to the tube dislodgment and blockage.

Conclusion

This study concludes that PC, using both laparoscopic and US-guided techniques, can serve as an interim as well as a definitive measure, particularly in patients who are at high risk for anesthesia and the procedure itself and have multiple comorbidities.

## Introduction

Acute cholecystitis (AC) refers to the inflammation of the gall bladder and is one of the most common emergency surgical presentations. In the UK, approximately 15% of the population is estimated to have gallstones [1]. It is anticipated that around 20% of patients with gallbladder stones will develop inflammation of the gallbladder [2]. Laparoscopic cholecystectomy (LC) is considered the definitive management of AC and is associated with low morbidity and good outcomes in young and otherwise healthy patients. However, the treatment of AC in high-risk individuals is still debatable, and because of a diminished physiological reserve, cholecystectomy in these patients can result in significant morbidity and mortality [3-5]. In addition to that, these patients are not considered suitable candidates for general anesthesia because of their reduced functional reserve [6].

Therefore, it is crucial to investigate other therapeutic options, such as laparoscopic and percutaneous catheter drainage, for this particular patient group. Surgical and radiological cholecystostomies have previously been used to treat high-risk, frail patients with multiple comorbidities. The first cholecystostomy was performed by Bobbs in 1867 [7]. It is believed to be an effective, minimally invasive, and safer approach to treat patients who are very ill due to ongoing sepsis, have failed medical treatment with antibiotics, or are generally high risk due to their multiple co-morbidities [8]. Although the least invasive approach is the management of such patients with a percutaneous image-guided cholecystostomy, however, in smaller district general hospitals, the lack of availability of radiological services out of hours renders the use of the laparoscopic approach a suitable alternative [9].

In addition to the above, the COVID-19 pandemic has also resulted in a significant increase in waiting lists. Even the delivery of cancer services was badly impacted and stopped in several trusts due to restricted surgical list capacity, limited access to critical care, and workforce reallocation. In the NHS, 4.5 million patients were waiting for elective care as of November 2019. An additional 1.2 million patients were projected to join that waiting list every three months of the pandemic, according to estimates [10]. This also impacts exploring appropriate alternative approaches to handle benign gall bladder surgery moving forward in both an effective and timely manner, given the imminent waiting list issue and ongoing strain on the healthcare system caused by COVID-19.

The aim of the study is to evaluate the outcomes of laparoscopic in comparison to percutaneous image-guided cholecystostomy as temporary or definitive management in high-risk patients admitted with AC in a district general hospital.

## Materials and methods

This retrospective study was conducted at the Royal Albert Edward Infirmary, which is a district general hospital based in Wigan, UK. A proposal was sent for approval and was registered with the research and audit department of Wrightington, Wigan and Leigh NHS Foundation Trust, Wigan, UK, before commencing. Data of all the patients admitted under the surgical team between January 2017 and December 2022 who were critically ill and underwent laparoscopic and radiological cholecystostomies due to ongoing sepsis or admitted with multiple comorbidities was retrieved using a coding system employed at our hospital, and the medical notes were thoroughly reviewed. Patients were categorized into two groups, group A and group B, based on the cholecystostomy procedures performed: laparoscopic and ultrasound (US)-guided, respectively. Group A comprised patients undergoing laparoscopic cholecystostomies, while group B included those undergoing US-guided procedures so that a comparative analysis of outcomes between the two techniques, considering factors such as resource availability and procedural feasibility can be made.

Inclusion criteria included patients who underwent laparoscopic or radiological cholecystostomies for AC due to being high-risk with multiple comorbidities, those who failed medical management with antibiotics, or those who presented with sepsis. All the patients who had hepato-pancreato-biliary (HPB) malignancy and those who underwent open cholecystostomies or had abdominal ascites at the time of presentation were excluded from the study.

Medical records of all the included patients were analyzed, and data was collected regarding the demographic details of the patients, American Society of Anesthesiologists (ASA) grades, success rates of both procedures as temporary or definitive management, duration of hospital stay, 30-day and 1-year mortality after the procedure, timing of the procedure (day or night), and long-term complications after the procedure related to cholecystostomy tube dislodgment or blockage. The data of all the patients was recorded and analyzed using the Microsoft Excel software (Microsoft, Redmond, US). Statistical analysis was performed using the chi-square test for categorical variables. A significance level of 0.05 was utilized, with statistical significance considered for p-values less than 0.05. The analysis was conducted to assess associations between categorical variables and determine the significance of differences between groups.

## Results

**Figure 1:**
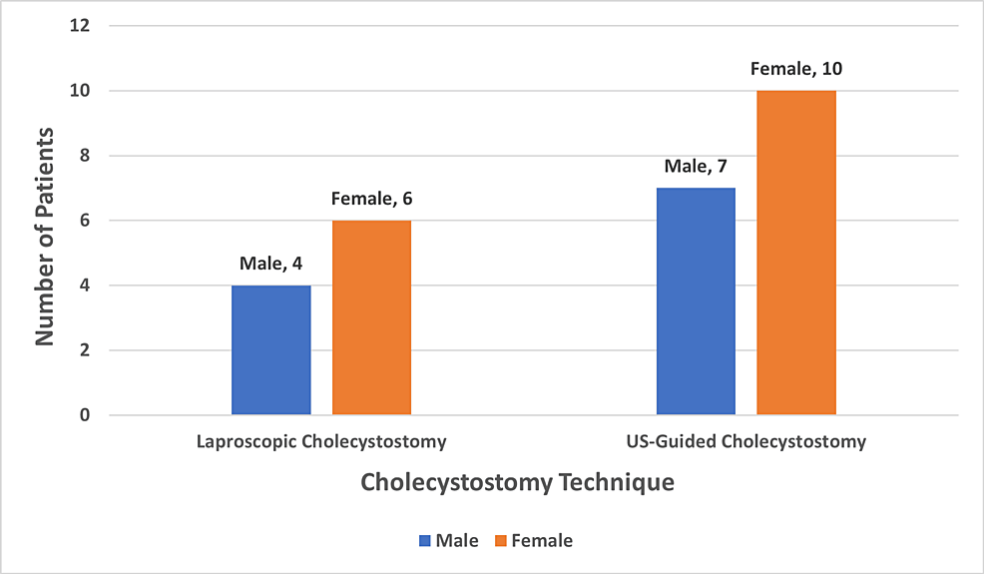
Gender distribution of the study participants US: Ultrasound

**Figure 2:**
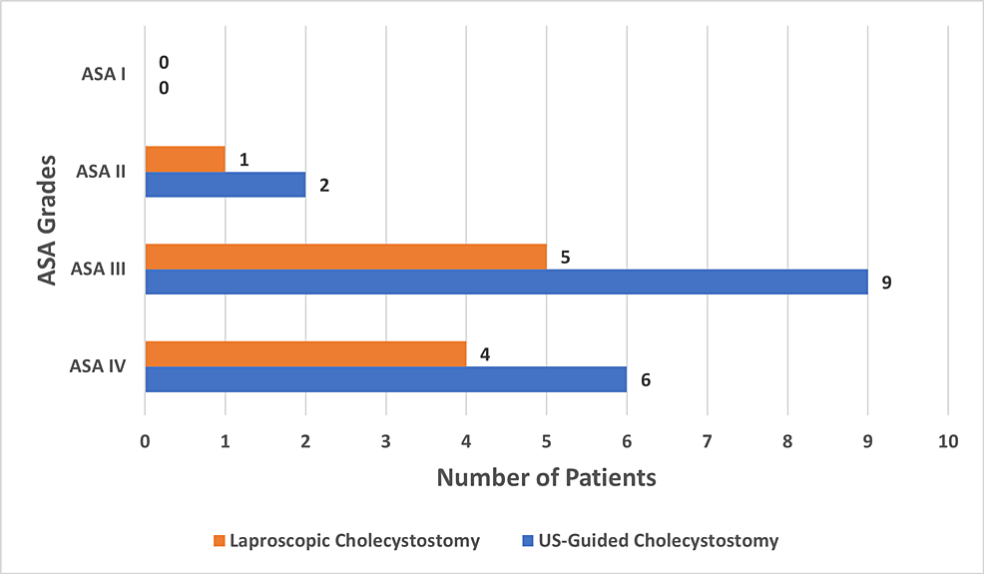
The participant data categorized on the basis of ASA levels ASA: American Society of Anesthesiologists; US: Ultrasound

A total of 27 patients who underwent laparoscopic and US-guided cholecystostomies were included in the study. Group A, consisted of 10 patients who underwent laparoscopic cholecystostomies, and group B, consisted of 17 patients who had US-guided cholecystostomies. Figure 1 shows the distribution of males and females in both study groups, and Figure 2 represents the ASA grading of the patients included in the study.

The mean age of the patients in group A was 66.7 as compared to 75.1 in group B. All the US-guided cholecystostomies were carried out during routine working hours, as our trust does not have out-of-hours radiology services available as compared to laparoscopic cholecystostomies which were evenly distributed between day and night. The majority of the patients who underwent the laparoscopic approach had their elective LC performed as compared to only 1/3rd of the US-guided population. The mean ASA score was similar between the two groups. However, patients undergoing US-guided procedures had a longer duration of hospital stay compared to those undergoing LCs. Both groups had comparable 30-day mortality rates, but US-guided patients had a higher one-year mortality rate. Tube dislodgement/blockage occurred more frequently in US-guided procedures compared to LCs.

The analysis revealed a significant disparity (p = 0.0004) in the timing of the procedure, with daytime US-guided procedures being contrasted with nighttime laparoscopic ones. Planning for elective cholecystectomy post-procedure revealed a significant difference (p = 0.0057), favoring a higher proportion of planned surgeries in the laparoscopic group. However, no significant differences were found in the duration of hospital stay (0.379), nor the 30-day mortality rate (p = 0.69). Although the one-year mortality rate did not reach statistical significance (p = 0.086), a trend towards higher mortality was observed in the US-guided cholecystostomy group. Table 1 shows the detailed outcomes of the study comparing the two types of approaches used as well as the combined outcomes of the total study population.

**Table 1:** Comparison of the laparoscopic vs US-guided PC + combined outcomes of the total study population US: Ultrasound; ASA: American Society of Anesthesiologists; LC: Laparoscopic cholecystectomy; PC: Percutaneous cholecystostomy

Outcome	Laparoscopic cholecystostomy Total Number (10) Number (percentage)	US-guided cholecystostomy Total Number (17) Number (percentage)	Combined laparoscopic and US-guided Total Number (27) Number (percentage)	p-value
Timing of the procedure	Day 3 (30%)	Day 17 (100%)	Day 20 (74%)	0.0004
	Night 7 (70%)	Night 0 (0%)	Night 7 (26%)	
Planned for elective LC	9 (90%)	6 (35%)	15 (55%)	0.0057
Mean ASA	2.5	2.7	2.6	0.305
Duration of hospital stay	9 days	18 days	13.5 days	0.379
30-day mortality	1 (10%)	1 (5.8%)	2 (7.4%)	0.69
1-year mortality	1 (10%)	7 (41.17%)	8 (29.6%)	0.086
Tube dislodgement/blockage	3 (30%)	8 (47%)	11 (40%)	0.143

## Discussion

AC, being one of the most common emergency surgical presentations, is responsible for a significant burden on the healthcare system. LC is considered the standard of care and mainstay of treatment for AC [11]. National Institute for Health and Care Excellence guidelines recommend that all patients with AC should have their LC within one week of diagnosis [12]. Realistically speaking, this target is very difficult to achieve, and there are only a handful of trusts in the UK with specialized HPB centers where index admission laparoscopic cholecystectomies are carried out. The situation has further worsened after the COVID-19 pandemic due to massive cancellations of surgical lists, thus creating more pressure on the NHS. This increasing burden of waiting lists has forced trusts to explore the option of weekend lists to help decrease these rising numbers [13].

In this situation, cholecystostomy can be a suitable temporary alternative or definitive management procedure, especially in high-risk patients. This is also supported by Tokyo 2013 guidelines, according to which urgent biliary draining via PC should be carried out for high-risk patients with grade II (moderate) or III (severe) AC [14]. This is also supported by a multi-level randomized controlled trial by Kortram et al., notably recognized as the CHOCOLATE trial [15].

The majority of the recent studies addressing PC as a treatment option for high-risk patients with AC are retrospective and have small sample sizes, which is also the case in our study. This is likely because of the infrequent use of PCs in the management of AC. Our study showed an overall 30-day mortality rate of 7.4%, which is in line with a systemic review conducted by Winbladh et al., which showed a mortality rate ranging between 4% and 12.7% [16]. Although this is higher than the mortality rates for patients who undergo LC, this could be explained by selection bias, as it is anticipated that patients managed with PC were in a poorer clinical condition secondary to their commodities than those managed with LC.

Complications in the form of drain dislodgment or blockage in our study were approximately 40%, which is almost double the one conducted by Kortram et al., which showed a complication rate of 22%, and almost four times the rate reported by Winbladh et al. [16,17]. One striking difference between our study and Kortram et al. was that all of their patients were provided with pamphlets detailing the care and management of PC tubes, and this lack of patient education may have played a significant role in this higher complication rate [17].

The mean hospital stay in our study was 13.5 days, which is slightly higher than reported by Kaya et al. but equivalent to the one outlined by Bhatia et al. [18,19]. A total of 15 of our patients underwent planned LC after PC; 2 of them died within 30 days of the procedure, and 10 patients had PC as a definitive procedure because they were surgically very high-risk secondary to their significant comorbidities. Because our hospital does not support out-of-hours radiology service, none of our patients in group B underwent PC out of hours as compared to the seven patients in group A who had their PC done at night time. The limitations of this study include its retrospective design, small sample size limiting the statistical power and generalizability of the results, single-center district general experience restricting the external validity of the findings, and the absence of a control group. Moreover, reliance on the retrospective data collection methods, coupled with limited follow up weakens the completeness and accuracy of the data.

## Conclusions

Our study concludes that PC, both by laparoscopic and ultrasound guidance methods, is a viable, minimally invasive option for the management of AC, especially in high-risk patients with multiple comorbidities who are very high-risk for both the operation and anesthesia. It has a low risk of complications and could serve as an effective interim measure before a carefully planned elective cholecystectomy as well as a definitive option for patients who are deemed completely unfit for surgical operation.

## References

[1] Shaffer EA (2005). Epidemiology and risk factors for gallstone disease: has the paradigm changed in the 21st century?. Curr Gastroenterol Rep.

[2] Strasberg SM (2008). Clinical practice. Acute calculous cholecystitis. N Engl J Med.

[3] Ambe PC, Weber SA, Christ H, Wassenberg D (2015). Primary cholecystectomy is feasible in elderly patients with acute cholecystitis. Aging Clin Exp Res.

[4] Fukami Y, Kurumiya Y, Mizuno K, Sekoguchi E, Kobayashi S (2014). Cholecystectomy in octogenarians: be careful. Updates Surg.

[5] Nikfarjam M, Yeo D, Perini M (2014). Outcomes of cholecystectomy for treatment of acute cholecystitis in octogenarians. ANZ J Surg.

[6] Wahlen BM, De GA (2021). Anesthetic concerns in advanced age undergoing emergency surgery. Emergency General Surgery in Geriatrics.

[7] Sparkman RS (1967). Bobbs centennial: the first cholecystotomy. Surgery.

[8] Riall TS, Zhang D, Townsend CM Jr, Kuo YF, Goodwin JS (2010). Failure to perform cholecystectomy for acute cholecystitis in elderly patients is associated with increased morbidity, mortality, and cost. J Am Coll Surg.

[9] Han SP (2016). Laparoscopic cholecystostomy as an alternative to open cholecystectomy and percutaneous cholecystostomy in a rural setting. HPB.

[10] Macdonald N, Clements C, Sobti A, Rossiter D, Unnithan A, Bosanquet N (2020). Tackling the elective case backlog generated by Covid-19: the scale of the problem and solutions. J Public Health (Oxf).

[11] Papi C, Catarci M, D'Ambrosio L, Gili L, Koch M, Grassi GB, Capurso L (2004). Timing of cholecystectomy for acute calculous cholecystitis: a meta-analysis. Am J Gastroenterol.

[12] https://www.nice.org.uk/guidance/qs104/chapter/quality-statement-1-acute-cholecystitis.

[13] Clifford RE, Rajput K, Naing CY, MacDonald K, Pantak T, Kaul A (2022). Reducing waiting lists for laparoscopic cholecystectomy: an intensive approach to aid COVID-19 recovery. Eur Surg.

[14] Okamoto K, Takada T, Strasberg SM (2013). TG13 management bundles for acute cholangitis and cholecystitis. J Hepatobiliary Pancreat Sci.

[15] Kortram K, van Ramshorst B, Bollen TL (2012). Acute cholecystitis in high risk surgical patients: percutaneous cholecystostomy versus laparoscopic cholecystectomy (CHOCOLATE trial): study protocol for a randomized controlled trial. Trials.

[16] Winbladh A, Gullstrand P, Svanvik J, Sandström P (2009). Systematic review of cholecystostomy as a treatment option in acute cholecystitis. HPB (Oxford).

[17] Abdelsaid K, Hassan M, Jayasankar B, Jeilani M, Ali H, Abdul Aal Y (2023). Percutaneous cholecystostomy in severe acute cholecystitis: an observational study from a single Institute. Cureus.

[18] Kaya C, Bozkurt E, Ömeroğlu S (2018). Is interval cholecystectomy necessary after percutaneous cholecystostomy in high-risk acute cholecystitis patients?. Sisli Etfal Hastan Tip Bul.

[19] Bhatia M, Thomas B, Azir E (2023). Percutaneous cholecystostomy to manage a hot gallbladder: a single center experience. Cureus.

